# Prevalence of OXA-Type β-Lactamase Genes among Carbapenem-Resistant *Acinetobacter baumannii* Clinical Isolates in Thailand

**DOI:** 10.3390/antibiotics9120864

**Published:** 2020-12-03

**Authors:** Krit Thirapanmethee, Thayapa Srisiri-a-nun, Jantana Houngsaitong, Preecha Montakantikul, Piyatip Khuntayaporn, Mullika Traidej Chomnawang

**Affiliations:** 1 Department of Microbiology, Faculty of Pharmacy, Mahidol University, Bangkok 10400, Thailand; krit.thi@mahidol.ac.th (K.T.); thayapa.sir@mahidol.ac.th (T.S.-a.-n.); piyatip.khn@mahidol.ac.th (P.K.); 2 Department of Pharmacy, Faculty of Pharmacy, Mahidol University, Bangkok 10400, Thailand; jantana.hon@mahidol.ac.th (J.H.); preecha.mon@mahidol.ac.th (P.M.)

**Keywords:** resistance mechanism, carbapenem, carbapenemase, oxacillinase, multidrug resistance

## Abstract

Carbapenem-resistant *Acinetobacter baumannii* (CRAB) is a critical health concern for the treatment of infectious diseases. The aim of this study was to investigate the molecular epidemiology of CRAB emphasizing the presence of oxacillinase (OXA)-type β-lactamase-encoding genes, one of the most important carbapenem resistance mechanisms. In this study, a total of 183 non-repetitive CRAB isolates collected from 11 tertiary care hospitals across Thailand were investigated. As a result, the *bla*oxa-51-like gene, an intrinsic enzyme marker, was detected in all clinical isolates. The *bla*oxa-23-like gene was presented in the majority of isolates (68.31%). In contrast, the prevalence rates of *bla*oxa-40/24-like and *bla*oxa-58-like gene occurrences in CRAB isolates were only 4.92% and 1.09%, respectively. All isolates were resistant to carbapenems, with 100% resistance to imipenem, followed by meropenem (98.91%) and doripenem (94.54%). Most isolates showed high resistance rates to ciprofloxacin (97.81%), ceftazidime (96.72%), gentamicin (91.26%), and amikacin (80.87%). Interestingly, colistin was found to be a potential drug of choice due to the high susceptibility of the tested isolates to this antimicrobial (87.98%). Most CRAB isolates in Thailand were of ST2 lineage, but some belonged to ST25, ST98, ST129, ST164, ST215, ST338, and ST745. Further studies to monitor the spread of carbapenem-resistant OXA-type β-lactamase genes from *A. baumannii* in hospital settings are warranted.

## 1. Introduction

*Acinetobacter baumannii* is a Gram-negative coccobacillus that has the ability to easily acquire antibiotic resistance and to persist in hospital environments [1]. This organism is considered an opportunistic pathogen responsible for nosocomial infections, especially in intensive care units [2]. *A. baumannii* commonly causes bacteremia, nosocomial-acquired pneumonia or ventilator-associated pneumonia, catheter-related infections, meningitis, peritonitis, skin and wound infections, urinary tract infections, and endocarditis [3]. The ability to survive in dry or moist conditions at various pH levels and temperatures renders it able to grow in the hospital environment [1].

*A. baumannii* is one of the ESKAPE pathogens, along with *Enterococcus faecium, Staphylococcus aureus, Klebsiella pneumoniae, Pseudomonas aeruginosa,* and *Enterobacter* spp., which are responsible for the majority of nosocomial infections and are capable of “escaping” the bactericidal activity of antimicrobial agents [4,5]. *A. baumannii* can develop resistance to many classes of commonly used antimicrobial agents [6,7]. The emergence of carbapenem-resistant *A. baumannii* (CRAB), multidrug-resistant (MDR) *A. baumannii*, or even extensively drug-resistant (XDR) *A. baumannii* has been progressively increasing globally over the last decade [8,9].

Carbapenems have long been considered as a last resort to treat infections caused by MDR Gram-negative bacteria, but recently, carbapenem resistance has been increasingly common in *A. baumannii* [10]. Several resistance mechanisms of *A. baumannii* against carbapenems have been reported, including antimicrobial-inactivating enzymes, efflux pump, loss of the CarO outer membrane porin, and decreased target access [3,11,12]. One of the most important carbapenem resistance mechanisms is the production of class D β-lactamases (oxacillinase; OXA). This group of enzymes can hydrolyze oxacillin and the third-generation cephalosporins, but possesses weak activity against carbapenems [13]. OXA-producing *A. baumannii* was first reported in 1993 from the blood culture of a patient at the Edinburgh Royal Infirmary in 1985 [14]. At present, several subtypes of OXA-type enzyme have been reported, such as OXA-23, OXA-40/24, OXA-48, OXA-51, OXA-58, OXA-143, and OXA143 [15,16]. These enzyme-coding genes can be detected on either chromosomes or plasmids. The presence of OXA-23 has been widely reported in clinical isolates in many countries [17,18,19]. OXA-23-like β-lactamase enzymes have been found in outbreak isolates collected in the UK, East Asia, and South America, while OXA-40/24-like β-lactamases were found in the United States and Europe [20,21,22]. Therefore, the aim of this study was to investigate the molecular epidemiology of CRAB, focusing on the spread of isolates with Class D β-lactamases. Multi-locus sequence typing (MLST) was also applied to investigate the sequence typing of clinical isolates from a multicenter collection in Thailand.

## 2. Results

### 2.1. Antimicrobial Susceptibility Pattern

A total of 183 CRAB isolates were collected from 11 tertiary care hospitals across Thailand during 2016–2017. All isolates were obtained from clinical samples from non-duplicated patients (Table 1). The *Acinetobacter* isolates were phenotypically identified by microbiological and biochemical methods. The molecular detection of the *bla*oxa-51-like gene revealed a 353 bp band in all clinical isolates, which preliminarily confirmed the identification of the clinical isolates as being *A. baumannii*. The class D *bla*oxa-51-like gene is chromosomally encoded and appears to be intrinsic to *A. baumannii* [23].

**Figure 1:**
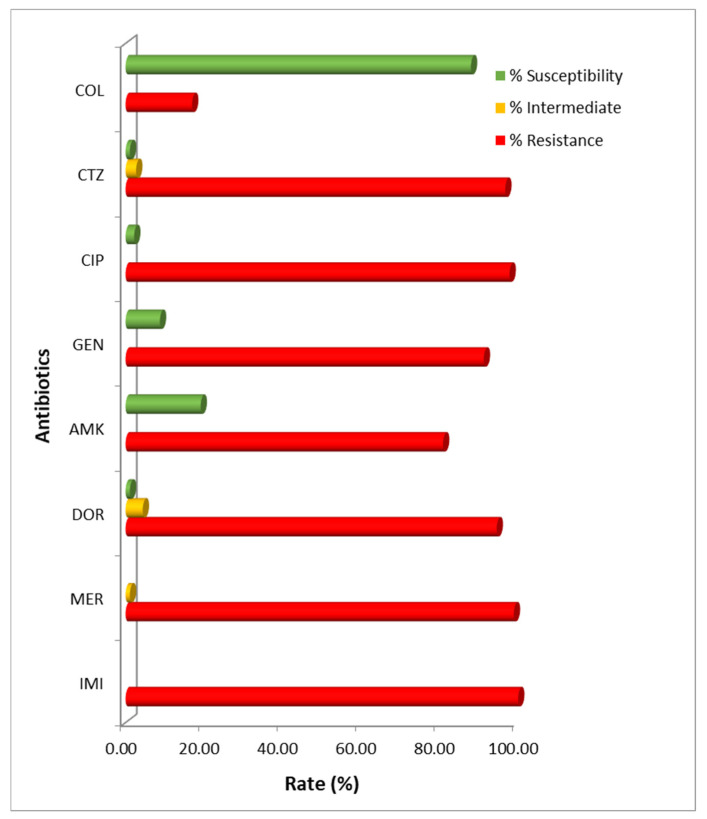
Antimicrobial susceptibility pattern of carbapenem-resistant *A. baumannii* isolates in Thailand. IMI: imipenem; MER: meropenem; DOR: doripenem; AMK: amikacin; GEN: gentamicin; CIP: ciprofloxacin; CTZ: ceftazidime; COL: colistin.

All clinical isolates were subjected to antimicrobial susceptibility testing utilizing the drugs currently used for *A. baumannii* treatment. The results demonstrated that all isolates were resistant to carbapenems, with 100% resistance to imipenem, followed by meropenem (98.91%) and doripenem (94.54%). High susceptibility was also observed with colistin (87.98%). Nevertheless, most of them showed high resistance rates to ciprofloxacin (97.81%), ceftazidime (96.72%), gentamicin (91.26%), and amikacin (80.87%) (Figure 1). The MIC_50_/MIC_90_ values were calculated for each antimicrobial agent, as follows: imipenem (32/64), meropenem (16/64), doripenem (16/64), ciprofloxacin (64/256), ceftazidime (512/>512), gentamicin (>512/>512), amikacin (>2048/>2048), and colistin (0.25/4), in micrograms per milliliter (Table 2). The MIC/MBC values of the control strain, *A. baumannii* ATCC 19606, were also examined for each antimicrobial agent: imipenem (≤1/≤1), meropenem (≤1/≤1), doripenem (≤1/≤1), ciprofloxacin (≤1/≤1), ceftazidime (16/16), gentamicin (16/16), amikacin (32/32), and colistin (2/2), in micrograms per milliliter.

### 2.2. Distribution of OXA-Type Carbapenemases

**Figure 2:**
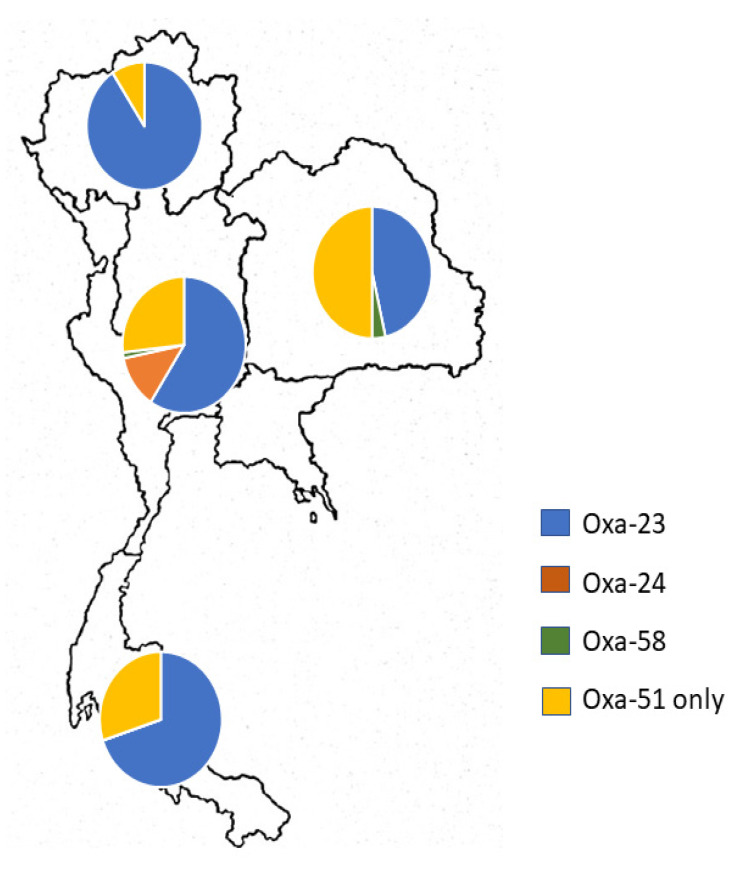
Geographical distribution of oxacillinase (OXA)-type carbapenemase-encoding genes present in carbapenem-resistant *A. baumannii* isolates from across Thailand.

All 183 non-repetitive CRAB isolates were further investigated for the presence of OXA-type carbapenemase-encoding genes. The PCR results showed that 68.31% of the clinical isolates (125/183) were positive for the *bla*oxa-23-like gene. Geographically, *bla*oxa-23-like-carrying CRAB isolates were predominantly distributed in the northern region (90.74%), followed by the southern (70%), central and metropolitan (59.15%), and eastern (46.43%) regions of Thailand (Figure 2).

In contrast, the prevalence rates of the *bla*oxa-40/24-like and *bla*oxa-58-like genes were only 4.92% and 1.09% in CRAB isolates, respectively. It was noteworthy that all *bla*oxa-40/24-like-carrying CRAB isolates were collected from the central region. There were only two *bla*oxa-58-like-carrying CRAB isolates from the central and southern regions of Thailand. Interestingly, concomitant existence of carbapenem-hydrolyzing class D β-lactamases (excluding the intrinsic OXA-51-like gene) was found in 10 isolates. Co-existence of the *bla*oxa-23-like and *bla*oxa-40/24-like genes was detected in nine isolates, while only one isolate carried the *bla*oxa-23-like and *bla*oxa-58-like genes. It was remarkable that these isolates simultaneously carried triple OXA carbapenemase genes, including the *bla*oxa-51-like gene. *A. baumannii* ATCC 19606, a standard control strain, displayed only the intrinsic *bla*oxa-51-like gene.

### 2.3. Molecular Typing by Sequence Type (ST) Analysis

The sequence types (STs) of the CRAB isolates were determined according to the Institute Pasteur MLST scheme. The results demonstrated that most CRAB isolates in Thailand were of ST2 lineage. There were also some isolates belonging to ST25, ST98, ST129, ST164, ST215, ST338, and ST745. In addition, ST2 was the most dominant type for *bla*oxa-23-like-carrying CRAB isolates. Besides ST2, ST338, and ST745 were also represented among the *bla*oxa-40/24-like-carrying CRAB isolates, while an isolate with the OXA-58-like gene belonged to ST164.

## 3. Discussion

The increasing incidence of CRAB infections is becoming a pivotal concern for public health since the carbapenem drug group is considered the last resort for the treatment of severe infections caused by *A. baumannii* [10]. Notably, the World Health Organization (WHO) lists CRAB in the critical priority category according to the urgency of their need for new antimicrobial drugs [24]. Moreover, high incidence rates of CRAB infections have been reported in Southeast Asia. *A. baumannii* is naturally resistant to many antimicrobial drugs and is well-recognized in the acquisition of antibiotic-resistant genes. The increasing resistance rate of this particular microorganism is considered a public health threat due to the limited treatment options. Effective treatment of CRAB infection is now based on colistin, tigecycline, or sulbactam in combination with ampicillin [25,26]. This study revealed that most CRAB isolates from Thailand were still susceptible to colistin, although high resistance rates were observed for ciprofloxacin, ceftazidime, gentamicin, and amikacin. The MIC_50_/MIC_90_ values of these drugs were exceedingly high (>4-fold MIC breakpoints), except for those of colistin, indicating more complicated treatment by the currently used drugs. This was possibly due to the multiple resistance mechanisms commonly found in *A. baumannii*.

In general, several mechanisms are involved in carbapenem resistance, such as the production of carbapenem-hydrolyzing β-lactamases or carbapenemases, reduced permeability, and efflux pump. Among these, carbapenemase production is the most effective mechanism [27]. Therefore, CRAB isolates from a multicenter collection in Thailand were investigated for the prevalence of class D β-lactamases in this study. Here, we report that the *bla*oxa-23-like gene was predominantly present in Thailand (68.31%). These results are in agreement with those of previous studies which described the spread of OXA-23-producing *Acinetobacter* isolates in various locations worldwide. In China, about 80.6% of CRAB isolates carried the *bla*oxa-23 gene [28]. In addition, the predominant OXA group associated with carbapenem resistance across India was *bla*oxa-23-like (97%) [29]. In the Middle Eastern region, including Egypt, Qatar, and the United Arab Emirates, the *bla*oxa-23-like gene of class D β-lactamases was mainly identified among isolated *A. baumannii* strains [30,31]. High prevalence of the *bla*oxa-23-like gene (82.6%) was also reported among all *A. baumannii* isolates, and this gene was detected in 79.1% of CRAB isolates, from hospitals in some regions of Thailand during 2013–2015 [32]. Our study has shown that the *bla*oxa-23-like gene is still the most widely spread drug-resistance gene in CRAB, while the presence of other OXA-type β-lactamase genes is significantly low. The opposite trend has been observed for the occurrence of the *bla*oxa-40/24-like and *bla*oxa-58-like genes in Thai CRAB isolates, with rates of only 4.92% and 1.09%, respectively.

Despite the increasing prevalence of *A. baumannii* in hospital settings, little is known about which genomic components contribute to the clinical presentation of this pathogenic bacterium. Multi-locus sequence typing is highly discriminative and has been a powerful tool for epidemiological studies globally. MLST has been applied to characterize and monitor clinically important bacterial pathogens, including *A. baumannii* [33]. Since data on the sequence types (STs) of CRAB in Thailand are limited, CRAB isolates were examined to determine the most dominant type in this investigation. Here, we report that the most prevalent sequence type among CRAB in Thailand was ST2. There were some isolates belonging to ST25, ST98, ST129, ST164, ST215, ST338, and ST745. The sequence type distributions of *A. baumannii* in Asia have been published in many reports. In India, the predominant ST was ST848 (20%), followed by ST451 (12%), ST195 (7%), and other less common STs [29]. A similar ST distribution to the Indian clinical isolates was reported in extensively drug-resistant *A. baumannii* isolates in Iran [34]. However, none of this study’s isolates had STs similar to those of Indian and Iranian isolates. In Asia, ST2 has been described as the most prevalent sequence type among CRAB in China and Lebanon [28,35]. ST2 belongs to CC2 in the Pasteur scheme and corresponds to CC92 in the Oxford scheme and international clone II, as previously identified [36]. CC92 was reported to be the majority clone of CRAB isolates in India [37]. Similar to the results of a study in Malaysia, most of the isolates were grouped under the CC92 clonal complex [38]. It was noteworthy that the predominant ST in Malaysia was ST195, but it was ST2 in Thailand. Even though the sample numbers of previous studies were quite limited, they still reflected the distribution of CC92 in the Asian region.

## 4. Materials and Methods

### 4.1. Bacterial Collection and Identification

A total of 183 CRAB isolates were collected from 11 tertiary care hospitals across Thailand during 2016–2017. All *A. baumannii* isolates were obtained from clinical samples (sputum, urine, pus, tissue, or blood) from non-duplicated patients. Geographically, all isolates were collected from three hospitals in the central region, three hospitals in the northern region, three hospitals in the eastern region, and two hospitals from the southern region. *A. baumannii* ATCC 19606 was obtained from the American Type Culture Collection (ATCC), VA, USA. The Acinetobacter isolates were identified using conventional microbiological and biochemical methods. The samples were kept in glycerol stock and stored at −80 °C before use. The study was approved by the Ethical Review Committee, Faculty of Dentistry and Faculty of Pharmacy, Mahidol University (MU-DT/PY-IRB 2016/008.0404).

### 4.2. Antimicrobial Susceptibility Testing

Antimicrobial susceptibility testing for CRAB identification was carried out using disc diffusion and broth dilution methods with carbapenems (imipenem, meropenem, and doripenem) and other antimicrobial agents generally used for *A. baumannii* infections, namely, ceftazidime, ciprofloxacin, gentamicin, amikacin, fosfomycin, piperacillin/tazobactam, and colistin. *A. baumannii* isolates were grown in Mueller-Hinton broth (MH broth), and then diluted with MH broth to 0.5 McFarland before adding them into 96-well plates containing antibiotics in triplicate. Finally, the plate was kept at 37 °C for 18 h. The results were evaluated by the MIC values from the minimum concentration of drugs that gave no visible growth. The Clinical and Laboratory Standards Institute (CLSI) breakpoints were applied for susceptibility determination [39]. In this study, CRAB was defined as an isolate resistant to at least one agent among the carbapenems. MDR-*A. baumannii* was defined as an isolate resistant to ≥1 agent in ≥3 classes of antimicrobial categories among aminoglycosides, antipseudomonal carbapenems, antipseudomonal fluoroquinolones, antipseudomonal penicillin combined with β-lactamase inhibitors, extended-spectrum cephalosporins, folate pathway inhibitors, penicillin plus β-lactamase inhibitors, polymyxins, and tetracyclines [40].

### 4.3. Genotypic Determination of Carbapenemases

Genomic DNA from *Acinetobacter* species was prepared using the boiling method [41]. Quantification of the extracted DNA was determined by spectroscopy at 260 nm. The polymerase chain reaction (PCR) method was performed for amplification of *bla*oxa-23-like, *bla*oxa-24/40-like, *bla*oxa-51-like, and *bla*oxa-58-like *A. baumannii* isolates. The *bla*oxa-51-like gene, an intrinsic enzyme marker, was utilized as a reliable marker for the identification of *A. baumannii* according to the study by Turton and colleagues [23]. The specific primers and product sizes are shown in Table 3 [42]. The amplification process for the intrinsic OXA-23-like gene was initial denaturation at 94 °C for 5 min, 30 cycles of 94 °C for 25 s, 55 °C for 40 s, and 72 °C for 50 s, and a final elongation at 72 °C for 6 min. The amplification process for the intrinsic OXA-24/40-like and OXA-51-like genes was initial denaturation at 94 °C for 5 min, 30 cycles of 94 °C for 25 s, 58 °C for 40 s, and 72 °C for 50 s, and a final elongation at 72 °C for 6 min. The amplification process for the intrinsic OXA-58-like gene was initial denaturation at 94 °C for 5 min, 30 cycles of 94 °C for 25 s, 64 °C for 40 s, and 72 °C for 50 s, and a final elongation at 72 °C for 6 min.

### 4.4. Multi-Locus Sequence Typing Analysis

MLST was performed on CRAB isolates carrying either the *bla*oxa-23-like, *bla*oxa-24/40-like, or *bla*oxa-58-like gene. According to the Institute Pasteur protocol, the primers specific for seven housekeeping genes (*recA*, *gltA*, *fusA*, *cpn60*, *pyrG*, *rplB*, and *rpoB*) were used for PCR amplification, and PCR products were purified with a commercial kit according to the manufacturer’s instructions (Favorgen, Ping-Tung, Taiwan) [33]. Nucleotide sequencing was carried out by Bio Basic Asia Pacific Pte Ltd., Singapore. The nucleotide sequences were analyzed by comparing them to those in the Institute Pasteur database (https://pubmlst.org/abaumannii/).

## 5. Conclusions

Carbapenem resistance in *A. baumannii* occurs mainly as a result of the acquisition of OXA-type β-lactamase-encoding genes. The effect of the OXA β-lactamases in conferring carbapenem resistance has demonstrated a significant clinical impact on the ability to treat nosocomial infections. In this study, we report the recent situation covering CRAB isolates from multiple centers across Thailand. To date, the *bla*oxa-23-like gene, the first OXA-type β-lactamase gene to be identified from CRAB, still remains the most prevalent globally. The prevalence of the *bla*oxa-23-like gene was found to be relatively high in Thailand. The co-occurrence of two distinct carbapenemase-encoding genes in a single isolate was also detected in this study. In addition, the high occurrence rate of ST2 clinical isolates suggests that ST2, belonging to CC2 in the Pasteur scheme and CC92 in the Oxford scheme, could be an emerging lineage spreading carbapenem resistance in Thailand. Further studies are necessary to monitor the spread of carbapenem-resistant OXA-type β-lactamase genes from *A. baumannii* in hospital settings since they are becoming a significant cause of carbapenem resistance.

## Figures and Tables

**Table 1:** Specimen types from which carbapenem-resistant *Acinetobacter* *baumannii* isolates were recovered.

Isolation Site	No. of Isolates (%)
Sputum	147 (80.33)
Pus	17 (9.29)
Blood	8 (4.37)
Urine	9 (4.92)
Tissue	2 (1.09)
Total	183 (100)

**Table 2:** The antimicrobial susceptibility results for all 183 carbapenem-resistant *A. baumannii* (CRAB) isolates.

Antibiotics	MIC Range ^a^	MIC_50_^a^	MIC_90_^a^	Percentage (*n*)		
				Susceptible	Intermediate	Resistant
Carbapenems						
Imipenem	8–1028	32	64	0.00 (0)	0.00 (0)	100.00 (183)
Meropenem	4–256	16	64	0.00 (0)	1.09 (2)	98.91 (181)
Doripenem	0.125–256	16	64	1.09 (2)	4.37 (8)	94.54 (173)
Ciprofloxacin	0.125–>512	64	256	2.19 (4)	0.00 (0)	97.81 (179)
Ceftazidime	4–>512	512	>512	1.09 (2)	2.69 (5)	96.17 (176)
Gentamicin	0.5–>512	>512	>512	8.74 (16)	0.00 (0)	91.26 (167)
Amikacin	1–>2048	>2048	>2048	19.13 (35)	0.00 (0)	80.87 (148)
Colistin	0.0625–512	0.25	4	87.98 (152)	0.00 (0)	16.94 (31)

^a^ Values are presented in µg/mL.

**Table 3:** Specific primers used for PCR amplification of OXA carbapenemase genes.

Gene	Primer Sequences (5′ to 3′)				Product Size (bp)
	Forward	Tm (°C)	Reverse	Tm (°C)	
Class D					
oxacillinases					
*bla*oxa-23-like	GAT CGG ATT GGA	60	ATT TCT GAC CGC	56	501 bp
	GAA CCA GA		ATT TCC AT		
*bla*oxa-24/40-like	TAA TGC TTT GAT	60	AGT TGA GCG	58	246 bp
	CCC TTA AA		CAT CTT GG		
*bla*oxa-51-like	TAA TGC TTT GAT	58	TGG ATT GCA CTT	58	353 bp
	CGG CCT TG		CAT CTT GG		
blaoxa-58-like	AAG TAT TGG GGC	60	CCC CTC TGC GCT	64	599 bp
	TTG TGC TG		CTA CAT AC		
